# Higher reward value of starvation imagery in anorexia nervosa and association with the Val66Met *BDNF* polymorphism

**DOI:** 10.1038/tp.2016.98

**Published:** 2016-06-07

**Authors:** J Clarke, N Ramoz, A-K Fladung, P Gorwood

**Affiliations:** 1 Clinique des Maladies Mentales et de l'Encéphale (CMME), Hospital Sainte-Anne, Paris-Descartes University, Paris, France; 2 Centre of Psychiatry and Neuroscience, INSERM UMR 894, Paris, France; 3 Department of Psychiatry and Psychotherapy, University of Ulm, Ulm, Germany

## Abstract

Recent studies support the idea that abnormalities of the reward system contribute to onset and maintenance of anorexia nervosa (AN). Next to cues coding for overweight, other research suggest cues triggering the proposed starvation dependence to be pivotally involved in the AN pathogenesis. We assessed the characteristics of the cognitive, emotional and physiologic response toward disease-specific pictures of female body shapes, in adult AN patients compared with healthy control (HC) women. Frequency and amplitude of skin conductance response (SCR) in 71 patients with AN and 20 HC were registered during processing of stimuli of three weight categories (over-, under- and normal weight). We then assessed the role of the Val66Met *BDNF* polymorphism as a potential intermediate factor. AN patients reported more positive feelings during processing of underweight stimuli and more negative feelings for normal- and overweight stimuli. The SCR showed a group effect (*P*=0.007), AN patients showing overall higher frequency of the response. SCR within patients was more frequent during processing of underweight stimuli compared with normal- and overweight stimuli. The Met allele of the *BDNF* gene was not more frequent in patients compared with controls, but was associated to an increased frequency of SCR (*P*=0.008) in response to cues for starvation. A higher positive value of starvation, rather than more negative one of overweight, might more accurately define females with AN. The Met allele of the *BDNF* gene could partly mediate the higher reward value of starvation observed in AN.

## Introduction

Anorexia nervosa (AN) is defined by the persistent restriction of energy intake relative to requirements, leading to significantly low-body weight, an intense fear of gaining weight or becoming fat.^1^ AN has the highest mortality rate of all psychiatric disorders and is associated with severe and frequent comorbid psychiatric and somatic complications.^2, 3^ AN remains poorly understood and one of the main challenges is the identification of factors involved in the pattern of chronicity, relapse and severity.^4^ AN has a high genetic component with an heritability of about 0.7.^5^ As up to 63% of patients have a lack of recognition of the seriousness of the current low-body weight,^6, 7^ AN should be evaluated not only through the verbal expression of symptoms, but also through more reliable endophenotypes. The approach based on endophenotypes could indeed allow a better understanding of the physiopathology of this complex disorder and facilitate the identification of the genes involved. Endophenotypes may be more suitable for detecting risk genes, because they are genetically less complex than phenotypes (that is, related to fewer genes than phenotypes).^8^

Recent studies support the idea that AN could be the consequence of aberrant reward processing^9^ and, combined with exaggerated control, could involve neural circuits implicated in reward processing and compulsivity.^10^ Altered patterns of brain activity associated with emotional and reward processing tasks, related to more or less specific stimuli of the illness, have provided important information about the mechanisms underlying AN symptoms.^11^ It has been suggested that thinness could have a reward value in AN. Disturbance of the body image perception—classically described as a misconception and a major dissatisfaction of the own body—is a key diagnostic criterion in AN, which could be involved in the development, maintenance and relapse of the disorder.^12^

In two functional magnetic resonance imaging (fMRI) studies, Fladung *et al.*^13^ observed a specific reward pattern distinguishing patients with AN from healthy participants during self-referent processing of visual stimuli cuing for starvation in adult^13^ and in adolescent^14^ patients. During the task, AN patients reported most positive feelings while processing underweight stimuli and showed higher activation in the ventral striatum compared with controls. The ventral striatum is a brain region known for its major involvement in the reward circuit.^15, 16^ Nevertheless, the specificities of the emotional response to the female body shape (from starvation to overweight) remain insufficiently studied in AN, especially as the previous results are not really compatible with the proposition that an 'anhedonic profile' is characteristic of patients with AN.^11^ To resolve this paradox between 'anhedonic profile' and the 'hedonic profile' regarding high activation of the reward system and emotional response to starvation body shape in AN patients, it was more broadly proposed that AN could be linked to a global alteration of reward and emotional information processing, including the integration of cognitive and physiological components of emotion.^11, 17, 18^ The neurocognitive specificities of the response to starvation in AN could therefore potentially constitute an endophenotype.

We initially detected an excess of the Met allele in 1142 Caucasian patients with eating disorder (including AN) consecutively recruited from five European countries.^19^ Using a family-based association study in 453 trios, we replicated the excess of the Met66 allele in patients with AN, restrictive type.^20^ The role of the BDNF gene in AN could be related to its role in reward functions. Indeed, in a recent study analyzing the role of the BDNF Val66Met polymorphism in reward-related brain function in adolescents, a significantly lower BOLD response in the striatum was detected in Val/Val compared with Met allele carriers during reward anticipation,^21^ and BDNF Met66 carriers showed increased BOLD responses during anticipation of monetary losses in the ventral tegmental area–nucleus accunbens–medial prefrontral cortex (VTA–NAc–mPFC) circuit in healthy adult controls.^22^ Animal studies could support an important role of BDNF in rewarding processing, as increased BDNF levels within the ventral tegmental area induced an opiate dependent-like reward state^23^ and BDNF expression in the striatum explains part of voluntary alcohol consumption^24^ in rats. Furthermore, this functional Val66Met (rs6265) polymorphism has been associated with inter-individual human variations in reward processing.^25^

The aim of the present study was to assess the rewarding emotional response to starvation in patients with AN. Following the work of Fladung *et al.*^13^ and using their previously validated paradigm, we assessed the characteristics of the cognitive, emotional and physiologic response toward disease-specific pictures of female body shapes, in adult AN patients compared with healthy control (HC) women. We also investigated the possible contribution of the functional *BDNF* Val66Met polymorphism as a modulator of emotional response to starvation in AN.

## Materials and methods

### Participants

The sample included 71 adult females suffering from AN and 20 female HC participants, who agreed to participate in the study. Patients were recruited at the Clinique des Maladies Mentales et de l'Encéphale (CMME), a specialist care center for eating disorders (Hôpital Sainte-Anne, Paris, France), after entering specific treatment programs. All participants in the AN group were diagnosed with AN as outlined in the fifth edition of the Diagnostic and Statistical Manual of Mental Disorders.^1, 26^ The healthy comparison participants were matched to patients with AN according to sex, age and education level.

Controls were all voluntary women from the general population, friends of acquaintances of patients. They were initially contacted by phone and then recruited and assessed by the principal investigator. To be eligible as a control, participants had to have no past or current eating disorder. Exclusion criteria for all participants were the following: age <18, neurological disease, severe current somatic illness, hearing and visual impairments and current pregnancy.

All patients and controls reported Caucasian continental origins based on the country of birth of their four grandparents (inclusion criteria).

All participants gave their written informed consent to participate in the study, after a detailed explanation of its procedures. The protocol was approved by the regional ethic committee (Comité de Protection des Personnes).

Among AN patients, 36 (50.7%) were hospitalized at the time of assessment and 35 (49.3%) were outpatients. Thirty six (50.7%) patients had the diagnostic criteria of the restricting type, all others (*N*=35, 49.3%) having the binge eating/purging type. None of the participants had a diagnosis of schizophrenia, psychosis not otherwise specified, current major depressive episode, bipolar disorder or binge eating disorder. Concerning other psychiatric comorbidities, there were 10 (14.1%) patients with a lifetime diagnosis of social phobia, 9 (12.7%) with dysthymia, 16 (22.5%) with generalized anxiety disorder and 3 (4.2%) with obsessive-compulsive disorder. None of the participants had substance addiction, except for tobacco. Regarding psychotropic medication, 25 (35.2%) patients were treated with benzodiazepines, 28 (39.4%) with antidepressant treatment (SSRI or SNRI-type) and 17 (23.9%) with antipsychotic treatment. Twenty seven patients (38.0%) were not receiving any psychotropic medication. The 20 HCs declared that they have no neurological disorder, severe physical illness nor receiving any psychotropic medication.

### Clinical assessment

Participants completed a structured diagnostic interview using the Mini International Neuropsychiatric Interview.^27^ Psychotropic and non-psychotropic medications were also recorded.

Participants completed the Eating Attitude Test^28, 29^ and the Bulimic Investigatory Test, Edinburgh,^30^ assessing current, minimal and maximal body mass index (BMI) and amenorrhea. They also completed the Body Shape Questionnaire (BSQ)^31^ to measure concerns on body perception.

### Procedure of paradigm

#### Stimulus material

The experimental protocol was rolled out in line with the paradigm of visual stimulation previously used.^13^ The paradigm consisted of four successive sessions, with a total of 120 images being presented to the participant, in a randomized order. The 120 stimuli were computer-generated, representing a naked woman (height 165 cm), varying in BMI, with an underweight (BMI: 12–16 kg/ m^2^), normal weight (BMI: 19—23  kg/ m^2^) and overweight (BMI: 26—30  kg/ m^2^) figure. Images were presented in four different angles (15°, 25°, 35° and 45° of rotation to the right or left).

#### Tasks

Two tasks were proposed to the participant. The neutral control task was a 'categorization' task, which required the participants to estimate the weight of each visual stimulus from 1 to 4 on the numeric keyboard (corresponding to weight categories of 30, 45, 60 and 75 kg). The 'appraisal' task measured the appraisal of emotion in a self-referring way, answering the question 'How would you feel if you had the same shape?' Participants had to score their feeling during processing of each visual stimulus from 1 ('very bad') to 4 ('very good') on the numeric keyboard.

#### Physiological recording

Each participant was seated comfortably in a quiet room, with a 17-inch screen and recording material. For each participant, data were recorded 2 h after food intake (morning breakfast or midday lunch or snack only) to limit the potential effect on skin conductance recording of (1) postprandial satiety, (2) fasting and (3) caffeine intake. The autonomic measures were monitored using a 16-channel PowerLab system (AD Instruments, Bella Vista, NSW, Australia) connected to a PC. The bioloelectrical signals were filtered and amplified before being fed into the analog input connector of the PowerLab unit. Skin conductance was recorded with the ML116 GSR Amplifier (AD Instruments) of the 16-Channel PowerLabsystem, which provides a low and constant-voltage AC excitation. The dry electrodes were attached with a Velcro strap on the palmar surfaces of the distal phalange of the second and third fingers of the non-dominant hand. Measurements were displayed in μSv. In this study, there were two measures of electrodermal response (SCR): the average number of electrodermal responses, defined as a change of at least 0.04 μSv from baseline, and the amplitude of the responses in μSv. Skin conductance was scored as changes in conductance starting in the 1–4-s intervals after onset of each visual stimulus. Prior to measurements, electrodermal activity at baseline level was defined by a recording in the presence of a neutral visual stimulus.

### Genotyping

Genetic analyses were carried out on saliva samples (Oragene kit, DNA Genotek, Ottawa, ON, Canada), and DNA was extracted using standard methods. *BDNF* Val66Met polymorphism (SNP annotated rs6265) was performed using TaqMan SNP genotyping assay (Applied Biosystems, Life Technologies, Thermo Fisher Scientific, Waltham, MA, USA), detected on a real-time PCR DNA Engine OpticonTM 2 system (BIO-RAD, Hercules, CA, USA). Blanks and samples with known *BDNF* genotypes were taken along as quality controls during genotyping.

### Statistics

All statistical analyses were conducted using SPSS version 17.0 for Windows (IBM, Armonk, NY, USA). Clinical variables were analyzed using Student's two-sample *t*-tests to assess the effect of the groups. The normal distribution of all the data was first verified. Analyses of variance and pair-wise comparisons were conducted to compare intra- and intergroup effects on both explicit behavioral and implicit autonomic measures. Pair-wise comparisons were performed using a Bonferroni correction for multiple tests. Analyses of the relationships between *BDNF* Val66Met alleles and the SCR response frequency were performed using linear regression models.

## Results

### Recruited population and BDNF genetic polymorphisms

Ninety one participants were included in the protocol, with 71 AN patients and 20 HC (Table 1). Patients and HCs were not significantly different for age and education, all of them being female. Clinical characteristics of AN patients and controls are described in Table 1, with, as expected, lower minimum and current BMIs, and higher BSQ score for patients with AN. The distributions of the *BDNF* rs6265 genotypes were in Hardy–Weinberg Equilibrium in both groups (AN: *P*=0.900, HC: *P*=0.702). Twenty nine of 71 (40.8%) AN patients and 7 of 20 (35.0%) HC had at least 1 Met allele. AN and HC participants did not significantly differ in terms of genotype (*χ*^2^=0.2, *P*=0.637) or allele (*χ*^2^=0.0, *P*=1.000) distributions (Table 2).

### Categorization task

During the assessment of weight, AN patients estimated the weight of the underweight (AN 1.79±0.22 versus HC 1.6±0.3; *P*=0.005) and the normal weight (AN 3.2±0.3 versus HC 3.0±0.2; *P*=0.012) stimuli to be higher compared with HC. Groups did not differ concerning weight estimation of overweight stimuli (AN 3.8±0.2 versus HC 3.9±0.1; *P*=0.475) (Supplementary Figure 1).

### Subjective emotion ratings

**Figure 1:**
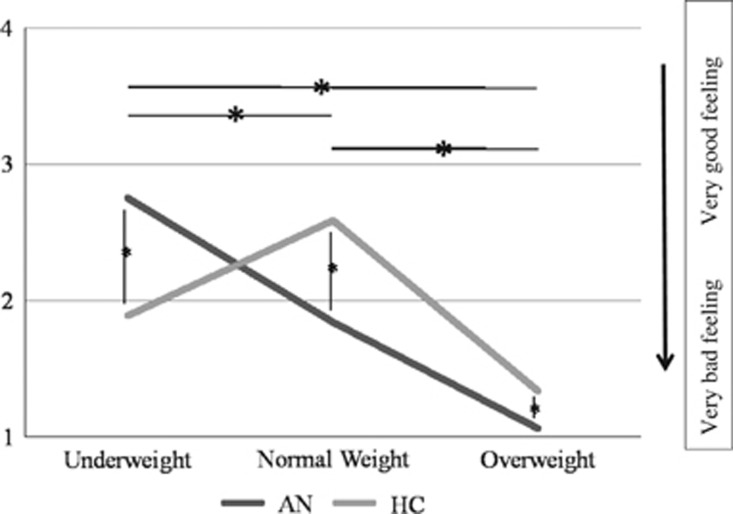
The BMI of the stimuli were ranging between 12 and 30. As initially proposed (Fladung *et al.*,^13^) these stimuli were gathered in three groups with underweight (BMI between 12 and 16), normal weight (17 and 23) and overweight (26 to 30). The feelings could be rated from very bad feelings (1) to very good feelings (4), with no in-between indications (ratings 2 and 3). *P<0.05, *inter and intra-group significant differences. AN, anorexia nervosa; BMI, body mass index; HC, healthy controls.

The rating scores of the 'appraisal' task were different between the two groups (F=39.6, degree of freedom (df)=2, *P<*0.001) (Table 3, Figure 1). Inter-group comparisons revealed that patients with AN, compared with HC, had higher positive scores for the underweight stimuli (2.7±0.5 versus 1.9±0.3; *P<*0.001), and lower positive scores for both the normal and the overweight stimuli (1.9±0.5 versus 2.6±0.5; *P<*0.001 and 1.1±0.1 versus 1.3±0.5; *P<*0.001, respectively). Interestingly, the difference of positive feelings during processing of underweight stimuli in patients compared with controls (average=0.8, s.d.=0.2) was significantly more pronounced than the difference of negative feelings during processing of overweight stimuli in patients compared with controls (average=0.2, s.d.=0.4) (*P<*0.001).

Intra-group comparisons revealed that patients with AN rated the processing of underweight stimuli more positive compared with normal- and overweight stimuli (*P<*0.001) (Figure 1). They also exhibited higher positive scores for normal weight stimuli compared with overweight stimuli (*P<*0.001). HC women had higher positive scores for normal weight stimuli compared with underweight (*P<*0.001) and overweight stimuli (*P<*0.001).

### Electrophysiological assessments

**Figure 2:**
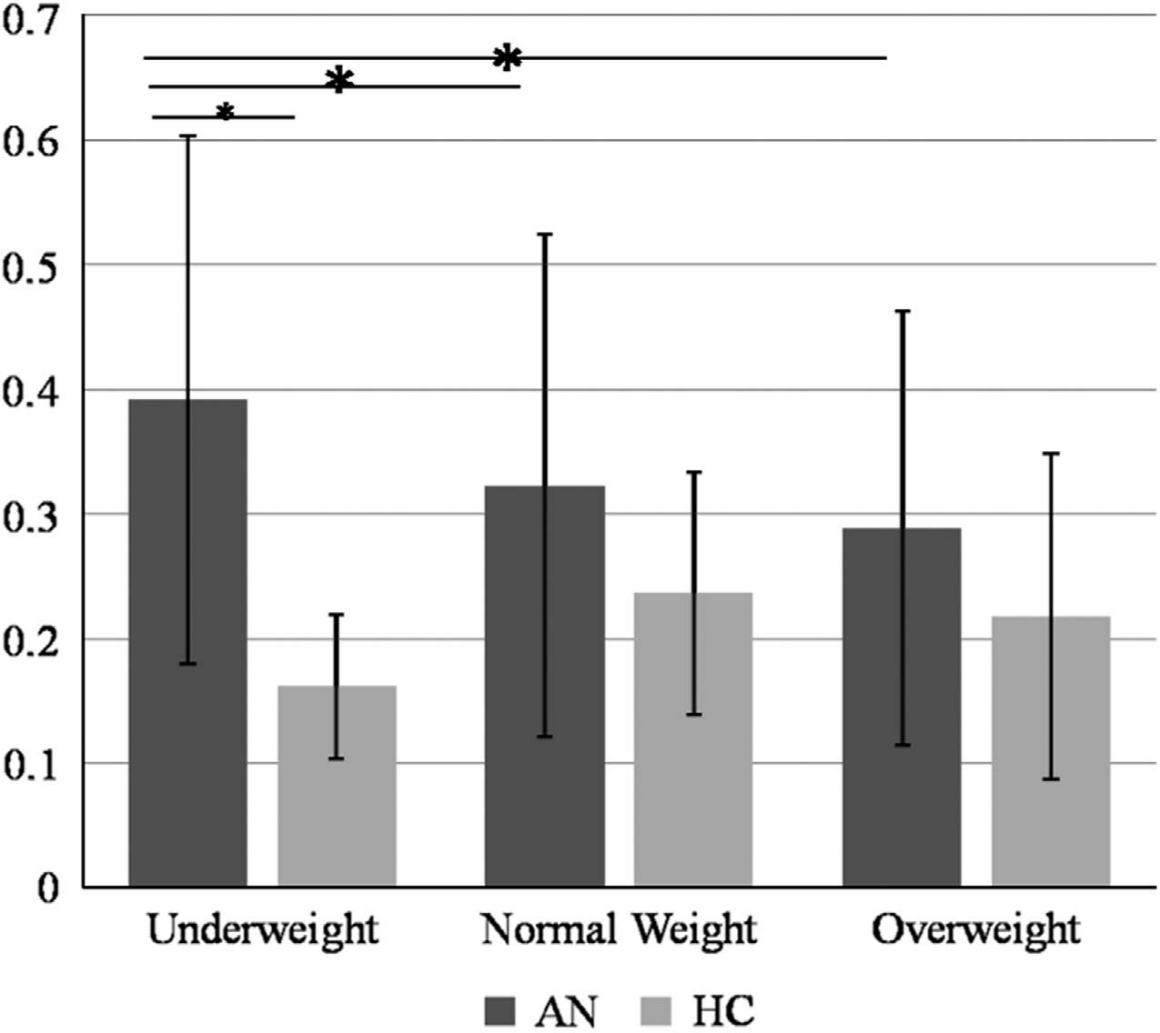
Average frequency of skin conductance reactivity to visual stimuli (underweight, normal weight and overweight) in anorexia nervosa (AN) and healthy control (HC) groups. *P<0.05. *inter and intra-group significant differences.

A significant group effect (F=7.6, df=2, *P*=0.007) was observed for the average frequency of SCR during processing of the visual stimuli. Thus, AN patients reacted more to all stimuli when compared with HC women. Inter-group comparisons revealed that patients with AN reacted more to underweight stimuli compared with HC participants (0.4±0.2 versus 0.2±0.1, F=23.0, *P<*0.001). This difference was not observed for normal (0.3±0.4 versus 0.2±1.0) and overweight (0.3±0.2 versus 0.2±0.1) stimuli (Table 3, Figure 2). Intra-group comparisons showed significant differences within the AN group. Patients reacted more to underweight stimuli compared with normal (*P<*0.001) and overweight stimuli (*P<*0.001). There was no significant difference in average frequency of SCR between the visual stimuli in the HC group (Figure 2).

We found significant differences in amplitude of SCR between the two groups (Table 3 and Supplementary Figure 2). AN patients had smaller response amplitude to underweight stimuli (0.5±0.4 versus 0.9±0.6, F=11.4 *P*=0.001) and overweight stimuli (0.5±0.4 versus 0.8±0.6, F=5.9, *P*=0.017) (Supplementary Figure 2). Intra-group comparisons showed no significant effect of the overall stimuli on the SC amplitude in patients and HC women. No difference on any physiological data was found for electrophysiological response to weight stimuli between AN patients with and without medication (Supplementary Table 1). Furthermore, no difference was found for emotional and electrophysiological response to underweight stimuli between AN patients with restrictive type versus those with the binge eating/ purging type (Supplementary Table 2).

### Genetics and emotional response

**Figure 3:**
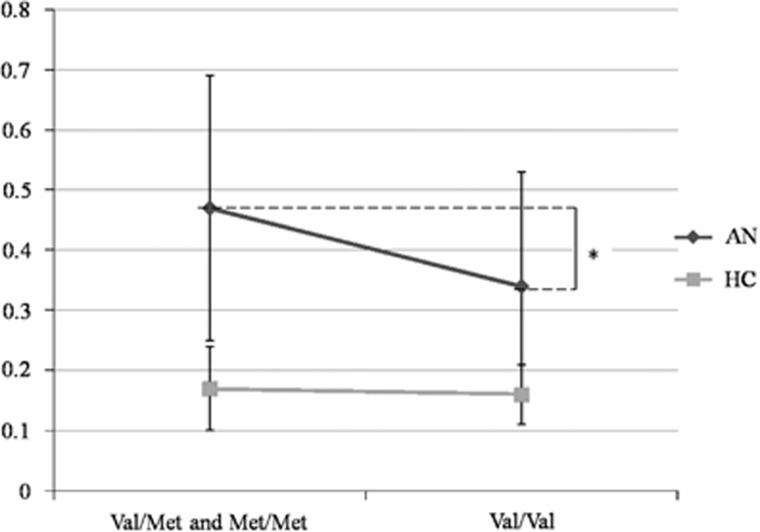
Average frequency of skin conductance reactivity to underweight in Val (Val/Val) and Met carriers (Val/Met and Met/Met) in patients with anorexia nervosa (AN) (*N*=71) and in healthy controls (HCs) (*N*=20). *P<0.05

We investigated the characteristics of the emotional response to processing of all stimuli (emotional and cognitive) in Met *BDNF* carriers compared with Val/Val participants, in both AN patients and the HC group (Table 3 and Figure 3). Present BMI, age and being or not hospitalized were not significantly associated with *BDNF* genotypes. No genotype effect was found on the average frequency of SCR while processing the three stimuli. Furthermore, no interaction between genotype and AN disease on the average frequency of SCR was found during processing of underweight stimuli (Supplementary Table 3).

In patients with AN subgroup analysis, Met carriers (Val/Met and Met/Met) had a significant increased frequency of SCR during processing of the underweight stimuli compared with Val homozygotes (0.5±0.2 versus 0.3±0.2, *P*=0.013) (Figure 3). In the HC group, there was no genotypic effect on the frequency of SCR (0.2±0.1 versus 0.2±0.1; *P*=0.588). We detected no significant correlation between clinical dimensions and subjective electrophysiological responses to underweight stimuli (Supplementary Table 4). We computed a linear regression model for the AN patients group to study the relationship between emotional response during processing of the underweight stimuli, the Met allele of the *BDNF* and different clinical criteria (age, current BMI, minimal BMI, psychotropic medication, subtype of AN, number of hospitalizations, duration of illness and severity score of the BSQ) (Supplementary Table 5). The average frequency of SCR during processing of underweight stimuli was still significantly associated with the presence of the Met allele (*P*=0.008) even when these clinical characteristics of AN were included in such multivariate analysis. The Met allele was associated with an increased SCR in response cues for starvation in AN patients but not in HC participants.

## Discussion

The aim of this study was to investigate the cognitive, rewarding emotional and electrophysiological response during processing of under-, normal- and overweight stimuli in patients with AN compared with HC. We also assessed the role of the functional *BDNF* Val66Met polymorphism as a modulator of the rewarding emotional response to thinness.

Subjective ratings in both groups showed that patients with AN had more positive feelings scores compared with HC when they were processing underweight stimuli, although patients overestimated this weight. Furthermore, patients with AN reported a higher positive value of processing of underweight stimuli, which was more pronounced than a negative value of processing overweight stimuli. SCR also differed significantly in frequency and amplitude in AN patients compared with HC participants and the SCR frequency was higher during the exposure to images of underweight bodies. In AN patients, a smaller amplitude of SCR could be linked to undernutrition or related physical condition. The higher frequency of SCR to underweight stimuli is associated with lower amplitude, it is therefore difficult to depict which one of these two aspects is more relevant to explain the salience of such specific visual stimuli. Last, the Met allele of the *BDNF* gene was specifically associated with an increased frequency of SCR during the exposure to thinness.

In a fMRI study proposing images of underweight bodies, the crucial brain region of the ventral striatum showed differential activity in participants with and without AN.^13^ Furthermore, another fMRI study performed in adolescents with AN and HCs reported more positive feeling in AN while facing underweight shapes. Relative to controls, underweight stimuli were already associated with greater activity of the ventral striatum, and processing of normal-weight stimuli elicited already reduced signaling, referring to the development of the disorder over time.^14^ Finally, the motivational salience of underweight shapes in patients with AN has been investigated in an electroencephalography study.^32^ Compared with normally developing adolescent girls, young AN patients presented a differential late positive potential pattern when viewing underweight women pictures. These results suggest an increased attention and motivation toward underweight stimuli in AN. The motivational salience of underweight stimuli in patients with AN seems to be particularly relevant and may promote pathological behaviors, maintaining starvation in patients. AN has often been described as the consequence of the fear to become fat or gain weight. A significant finding of this study is that patients with AN showed a pattern of emotional response, concerning both cognitive and electrophysiological aspects, showing a ‘preference for underweight' which was more specific than the ‘avoidance of overweight'. In addition to the cognitive measure of the emotional response, physiological measures were used to limit the ‘positive expectations' bias and social desirability. Further, the use of skin conductance measurements seems relevant for patients with introspective capacities that might be altered.

Association involving the *BDNF* Val66Met variant and AN in a large case–control study showed a significant role of the BDNF in eating disorders.^20^ In a study investigating 481 healthy adults, it has been reported that the Met/Met genotype carriers have a lower BMI compared with the Val/Met and Val/Val carriers.^33^ Nevertheless, a case–control study and a meta-analysis, including nine studies, have shown that the Val66Met variant was not associated with AN at detectable levels,^34^ a negative report which was recently confirmed by Boroska *et al.*^35^ who performed a genome-wide association study in 2907 cases with AN from 14 countries and 14 860 matched controls. The data in the literature are thus contradictory and the association between the Val66Met polymorphism and AN seems controversial. This finding could be due to the significant clinical heterogeneity of the disorder and could strengthen the interest of studying more specific biomarkers to further understand the condition. The Val66Met variant rs6265 is associated with a decreased secretion of mature BDNF.^36^ Altered BDNF levels, modulated by *BDNF* gene variability, are associated with the susceptibility to eating disorders, providing physiological evidence that BDNF could play a role in the development of AN and modulate eating behaviors and body weight regulations.^37^ This disturbed circulating levels of BDNF in patients with AN may help maintain starvation through its impact on the central pathways modulating reward.^38^ Here, we did not find any association between the Met allele of *BDNF* and AN, neither did we observe an effect of this allele and the 'feel task' assessments when facing different stimuli. More automatic (neuro-vegetative) tests of reward processing were indeed more informative.

Attempting to better understand the determinants of the emotional response to starvation in AN, we computed a linear regression model including the Met allele of the *BDNF* and many clinical characteristics of the illness. The SCR frequency during processing of underweight stimuli was significantly associated with the presence of the Met genotype but not with any of the clinical criteria of AN included in the model. The Met allele of the *BDNF* gene could partly mediate the observed higher reward value of starvation in patients with AN. The neurocognitive specificities of the response to thinness in AN may therefore constitute a candidate biological/endophenotypic marker.

Our study has several limitations. First, the size of 20 HCs can be considered as modest compared with the group of 71 AN patients. Nevertheless, the controls have been carefully screened for eating and other psychiatric disorders and matched to patients for different characteristics. Second, to study the specificities of the emotional response to starvation in AN, we included in this study women with heterogeneous clinical profiles. Weight status, psychiatric comorbid conditions or current medication could be important modulators of the emotional response in these patients, and we wanted to study the reward value of starvation in a representative population of patients encountered in clinical practice. In our study, 35 AN patients had a BMI ⩾17.5  kg/ m^2^, constituting a subgroup of patients fulfilling the criteria for subsyndromal AN (F50.1). All of them were treated for syndromic AN, but some had gained weight at the time of study entry. Even though we did not find a significant role of the present BMI, comparative electrodermal response to starvation in patients at both acute AN and after weight restoration would be an important topic to explore. During the task, patients overestimated the weight when they were processing underweight stimuli. The overall higher arousal might have influenced the stimulus-processing in patients with AN, and other non-body stimuli (from low to high arousal) could be included in the paradigm to confirm this observation. Finally, we were not able to control the participants' gaze direction during the execution of the paradigm. An eye-tracker processing may have been used to ensure that the emotional response was indeed due to the visual stimuli paradigm. When checking the coherence of results for each patient, as the paradigm was repeated more than once, we nevertheless did not find obvious inconsistencies.

There is an established model of reward processing that proposes three relevant components: liking (the hedonic aspect, you emphasize), wanting (the more action relevant aspect in terms of approaching behavior) and learning (referring to the development of processes).^36^ Thereby any attempt to disentangle the aspects of liking would have the fundamental requirement that these aspects are orthogonal to each other on both a conceptual and neurobiological level. However, this assumption does not appear to be appropriate, as a review on this topic concludes.^39^ Neurobiologicaly, the same neurochemical agent can be involved in processing of wanting and liking depending on its site of injection, which, for example, has been shown by microinjection of opioid agonists in the ventral striatum. In so-called 'hot-spots' liking and wanting reactions toward food in the rat were potentiated. Beyond these spots, however, only wanting, but not the liking reaction was enforced. Conceptually, if one interprets the liking aspect in a sense of assigning valence to a reward there is also a strong linkage between both aspects of 'liking' and 'approaching rewards' on that level. An interesting exception of this is 'irrational wanting', which can be induced in the animal through direct manipulations of the dopaminergic system via microinjections of amphetamine in the nucleus accumbens or drugs, activating catecholamine processing. This enforces the wanting but not the liking reaction, whereby the reward value reflects cue-triggered incentive-salience wanting which is sufficiently mediated by a subcortical neural reward system and does not need declarative goals or elaborative cognitive expectations activating cognitive cortical regions.^40^

In summary, a higher positive value of starvation, rather than more negative value of overweight, might more accurately define patients with AN. The Met allele of the *BDNF* gene could partly mediate the observed higher reward value of starvation in AN patients. Disturbed processes of emotional and reward response to starvation may thus promote strategies for maintaining pathological behaviors in patients with AN. Further studies are needed to explore the underlying neuronal mechanisms and the evolutionary terms of this response. It would help to better understand the mechanisms involved in the persistence of the disorder.

## Figures and Tables

**Table 1:** Demographic and clinical characteristics of patients with anorexia nervosa and healthy control women

	*Patients with anorexia nervosa (*N=*71)*			*Healthy controls (*N=*20)*					
*Characteristics*	*Mean*	*s.d.*	*Min–max*	*Mean*	*s.d.*	*Min–max*	t*-test*	*df*	P
Age (years)	27.4	9.1	18–54	27.2	9.2	18–53	0.08	89	0.94
Current body mass index (kg/ m^2^)	17.5	2.1	12.8–21.1	20.9	1.2	18.5–24	7.38	89	<0.001
Lowest lifetime body mass index (kg/ m^2^)	13.3	1.9	9.2–17.1	19.8	1.8	17.7–23.3	14.27	89	<0.001
Highest lifetime body mass index (kg/ m^2^)	21.7	4.4	16.7–46.1	22.2	2.3	18.3–26.3	0.64	89	0.52
Age at onset (years)	18.5	6.1	9.0–44	—	—	—	—	—	—
Duration of illness (years)	6.30	7.63	0.3–32	—	—	—	—	—	—
Body Shape Questionnaire scores	117.4	37.4	47–188	77.8	25.7	44–131	5.39	85	<0.001
Eating Attitude Test 40	41.4	25.8	3.0–95	6.4	4.0	3.0–15	14.5	65	<0.001
Bulimic Investigatory Test, Edinburg symptoms	14.9	8.2	1.0–28	1.4	1.3	0.0–4.0	20.98	65	<0.001
Bulimic Investigatory Test, Edinburg severity	4.5	5.2	0.0–21	0.5	1.0	0.0–2.0	4.39	65	0.04

Abbreviation: df, degree of freedom.

**Table 2:** Genetic distributions of the *BDNF* rs6265 variant of patients with anorexia nervosa and healthy control women

	*Patients with anorexia nervosa (*N=*71)*		*Healthy controls (*N=*20)*				
*Characteristics for the BDNF rs6265*	N	*%*	N	*%*	X*^2^*	*df*	P
Allele distribution for Met allele	32/142	22.5	9/40	22.5	0	1	1
Allele distribution for Val allele	110/142	77.5	31/40	77.5			
Genotype distribution for Met/Met and Val/Met	29/71	40.8	7/20	35	0.22	1	0.637
Genotype distribution for Val/Val	42/71	59.2	13/20	65			

Abbreviation: df, degree of freedom.

**Table 3:** Characteristics of the emotional and electrophysiological response to weight stimuli in anorexia nervosa and healthy controls groups

*Visual stimuli*	*Responses*	*Patients with anorexia nervosa (*N=*71)*						*Healthy controls (*N=*20)*						*Genotypes (all participants)*				*Current BMI effect*	*Group*	*Genotype*	*Interaction between genotype and group*
		*Met+ (*N=*29)*		*Met− (*N=*42)*		*All (*N=*71)*		*Met+ (*N=*7)*		*Met− (*N=*13)*		*All (*N=*20)*		*Met+*		*Met−*					
		*Average*	*s.d.*	*Average*	*s.d.*	*Average*	*s.d.*	*Average*	*s.d.*	*Average*	*s.d.*	*Average*	*s.d.*	*Average*	*s.d.*	*Average*	*s.d.*	P*-value*	P*-value*	P*-value*	P*-value*
Underweight	Feel task	2.8	0.4	2.7	0.5	2.7	0.5	2.0	0.2	1.8	0.3	1.9	0.3	2.6	0.8	2.5	0.6	0.002	<0.001	0.248	0.323
	SC+	0.5	0.2	0.3	0.2	0.4	0.2	0.2	0.1	0.2	0.1	0.2	0.1	0.4	0.2	0.3	0.2	0.079	<0.001	0.169	0.287
	SC amplitude	0.5	0.4	0.5	0.4	0.5	0.4	1.0	0.7	0.8	0.6	0.9	0.6	0.6	0.5	0.5	0.5	0.242	0.001	0.187	0.354
Normal weight	Feel task	1.7	0.6	1.9	0.5	1.9	0.5	2.5	0.6	2.6	0.4	2.6	0.5	1.9	0.7	2.1	0.6	0.269	<0.001	0.279	0.794
	SC+	0.4	0.5	0.3	0.3	0.3	0.4	0.3	0.1	0.2	0.4	0.2	1.0	0.4	0.4	0.3	0.3	0.018	0.347	0.294	0.938
	SC amplitude	0.8	0.1	0.5	0.6	0.6	0.4	0.7	0.4	0.6	0.4	0.7	0.6	0.8	1.3	0.5	0.5	<0.001	0.838	0.373	0.635
Overweight	Feel task	1.1	0.1	1.1	0.1	1.1	0.1	1.5	0.7	1.3	0.3	1.3	0.5	1.2	0.4	1.1	0.2	0.013	<0.001	0.044	0.074
	SC+	0.3	0.2	0.3	0.2	0.3	0.2	0.3	0.2	0.2	0.1	0.2	0.1	0.3	0.2	0.3	0.2	0.478	0.095	0.233	0.938
	SC amplitude	0.5	0.4	0.5	0.5	0.5	0.4	0.7	0.3	0.9	0.7	0.8	0.6	0.6	0.4	0.6	0.6	0.242	0.017	0.579	0.289

Abbreviations: BMI, body mass index; SC+, skin conductance response (average frequency); SC amplitude, skin conductance amplitude.

## References

[bib1] American Psychiatric Association Diagnostic and Statistical Manual of Mental Disorders, 5th edn (DSM-V). American Psychiatric Publishing: Arlington, VA, 2013.

[bib2] Papadopoulos FC, Ekbom A, Brandt L, Ekselius L. Excess mortality, causes of death and prognostic factors in anorexia nervosa. Br J Psychiatry 2009; 194: 10–17. 19118319 10.1192/bjp.bp.108.054742

[bib3] Arcelus J, Mitchell AJ, Wales J, Nielsen S. Mortality rates in patients with anorexia nervosa and other eating disorders. A meta-analysis of 36 studies. Arch Gen Psychiatry 2011; 68: 724–731. 21727255 10.1001/archgenpsychiatry.2011.74

[bib4] Steinhausen H-C. The outcome of anorexia nervosa in the 20th century. Am J Psychiatry 2002; 159: 1284–1293. 12153817 10.1176/appi.ajp.159.8.1284

[bib5] Gorwood P, Kipman A, Foulon C. The human genetics of anorexia nervosa. Eur J Pharmacol 2003; 480: 163–170. 14623359 10.1016/j.ejphar.2003.08.103

[bib6] Couturier J, Lock J. What is recovery in adolescent anorexia nervosa? Int J Eat Disord 2006; 39: 550–555. 16791851 10.1002/eat.20309

[bib7] Abbate-Daga G, Delsedime N, Nicotra B, Giovannone C, Marzola E, Amianto F et al. Psychosomatic syndromes and anorexia nervosa. BMC Psychiatry 2013; 13: 14. 23302180 10.1186/1471-244X-13-14 PMC3556145

[bib8] Gottesman II, Gould TD. The endophenotype concept in psychiatry: etymology and strategic intentions. Am J Psychiatry 2003; 160: 636–645. 12668349 10.1176/appi.ajp.160.4.636

[bib9] Kaye WH, Fudge JL, Paulus M. New insights into symptoms and neurocircuit function of anorexia nervosa. Nat Rev Neurosci 2009; 10: 573–584. 19603056 10.1038/nrn2682 PMC13038070

[bib10] Park RJ, Godier LR, Cowdrey FA. Hungry for reward: how can neuroscience inform the development of treatment for Anorexia Nervosa? Behav Res Ther 2014; 62: 47–59. 25151600 10.1016/j.brat.2014.07.007

[bib11] Keating C. Theoretical perspective on anorexia nervosa: the conflict of reward. Neurosci Biobehav Rev 2010; 34: 73–79. 19619579 10.1016/j.neubiorev.2009.07.004

[bib12] Halmi KA. Perplexities of treatment resistence in eating disorders. BMC Psychiatry 2013; 13: 292. 24199597 10.1186/1471-244X-13-292 PMC3829659

[bib13] Fladung A-K, Grön G, Grammer K, Herrnberger B, Schilly E, Grasteit S et al. A neural signature of anorexia nervosa in the ventral striatal reward system. Am J Psychiatry 2010; 167: 206–212. 19833790 10.1176/appi.ajp.2009.09010071

[bib14] Fladung A-K, Schulze UME, Schöll F, Bauer K, Grön G. Role of the ventral striatum in developing anorexia nervosa. Transl Psychiatry 2013; 3: e315. 24150224 10.1038/tp.2013.88 PMC3818005

[bib15] Groenewegen HJ, Trimble M. The ventral striatum as an interface between the limbic and motor systems. CNS Spectr 2007; 12: 887–892. 18163034 10.1017/s1092852900015650

[bib16] Van Kuyck K, Gabriëls L, Cosyns P, Arckens L, Sturm V, Rasmussen S et al. Behavioural and physiological effects of electrical stimulation in the nucleus accumbens: a review. Acta Neurochir Suppl 2007; 97: 375–391. 10.1007/978-3-211-33081-4_43 17691326

[bib17] Hatch A, Madden S, Kohn MR, Clarke S, Touyz S, Gordon E et al. Emotion brain alterations in anorexia nervosa: a candidate biological marker and implications for treatment. J Psychiatry Neurosci JPN 2010; 35: 267–274. 20598239 10.1503/jpn.090073 PMC2895157

[bib18] Keating C, Tilbrook AJ, Rossell SL, Enticott PG, Fitzgerald PB. Reward processing in anorexia nervosa. Neuropsychologia 2012; 50: 567–575. 22349445 10.1016/j.neuropsychologia.2012.01.036

[bib19] Ribasés M, Gratacòs M, Fernández-Aranda F, Bellodi L, Boni C, Anderluh M et al. Association of BDNF with anorexia, bulimia and age of onset of weight loss in six European populations. Hum Mol Genet 2004; 13: 1205–1212. 15115760 10.1093/hmg/ddh137

[bib20] Ribasés M, Gratacòs M, Fernández-Aranda F, Bellodi L, Boni C, Anderluh M et al. Association of BDNF with restricting anorexia nervosa and minimum body mass index: a family-based association study of eight European populations. Eur J Hum Genet 2005; 13: 428–434. 15657604 10.1038/sj.ejhg.5201351

[bib21] Nees F, Witt SH, Dinu-Biringer R, Lourdusamy A, Tzschoppe J, Vollstädt-Klein S et al. BDNF Val66Met and reward-related brain function in adolescents: role for early alcohol consumption. Alcohol 2015; 49: 103–110. 25650137 10.1016/j.alcohol.2014.12.004

[bib22] Peciña M, Martínez-Jauand M, Love T, Heffernan J, Montoya P, Hodgkinson C et al. Valence-specific effects of BDNF Val66Met polymorphism on dopaminergic stress and reward processing in humans. J Neurosci 2014; 34: 5874–5881. 24760847 10.1523/JNEUROSCI.2152-13.2014 PMC3996214

[bib23] Vargas-Perez H, Ting-A Kee R, Walton CH, Hansen DM, Razavi R, Clarke L et al. Ventral tegmental area BDNF induces an opiate-dependent-like reward state in naive rats. Science 2009; 324: 1732–1734. 19478142 10.1126/science.1168501 PMC2913611

[bib24] Bahi A, Dreyer JL. Striatal modulation of BDNF expression using microRNA124a-expressing lentiviral vectors impairs ethanol-induced conditioned-place preference and voluntary alcohol consumption. Eur J Neurosci 2013; 38: 2328–2337. 23601049 10.1111/ejn.12228

[bib25] Gasic GP, Smoller JW, Perlis RH, Sun M, Lee S, Kim BW et al. BDNF, relative preference, and reward circuitry responses to emotional communication. Am J Med Genet B Neuropsychiatr Genet 2009; 150B: 762–781. 19388013 10.1002/ajmg.b.30944 PMC7891456

[bib26] American Psychiatric Association Diagnostic and Statistical Manual of Mental Disorders, 4th edn (DSM-IV). Washington DC, USA,l 1994.

[bib27] Sheehan DV, Lecrubier Y, Sheehan KH, Amorim P, Janavs J, Weiller E et al. The Mini-International Neuropsychiatric Interview (M.I.N.I.): the development and validation of a structured diagnostic psychiatric interview for DSM-IV and ICD-10. J Clin Psychiatry 1998; 59: 22–33. 9881538

[bib28] Garner DM Eating Disorder Inventory-2 professional manual. Psychological Assessment Resources: Odessa, FL, USA, 1991.

[bib29] Garner DM, Garfinkel PE. The eating attitudes test: an index of the symptoms of anorexia nervosa. Psychol Med 1979; 9: 273–279. 472072 10.1017/s0033291700030762

[bib30] Henderson M, Freeman CP. A self-rating scale for bulimia. The “BITE''. Br J Psychiatry 1987; 150: 18–24. 3651670 10.1192/bjp.150.1.18

[bib31] Cooper PJ, Taylor MJ, Cooper Z, Fairburn CG. The development and validation of the Body Shape Questionnaire. Int J Eat Disord 1986; 6: 485–494.

[bib32] Horndasch S, Heinrich H, Kratz O, Moll GH. The late positive potential as a marker of motivated attention to underweight bodies in girls with anorexia nervosa. J Psychosom Res 2012; 73: 443–447. 23148812 10.1016/j.jpsychores.2012.09.020

[bib33] Gunstad J, Schofield P, Paul RH, Spitznagel MB, Cohen RA, Williams LM et al. BDNF Val66Met polymorphism is associated with body mass index in healthy adults. Neuropsychobiology 2006; 53: 153–156. 16707914 10.1159/000093341

[bib34] Brandys MK, Kas MJH, van Elburg AA, Ophoff R, Slof-Op't Landt MCT, Middeldorp CM et al. The Val66Met polymorphism of the BDNF gene in anorexia nervosa: new data and a meta-analysis. World J Biol Psychiatry 2013; 14: 441–451. 21936709 10.3109/15622975.2011.605470

[bib35] Boraska V, Franklin CS, Floyd JaB, Thornton LM, Huckins LM, Southam L et al. A genome-wide association study of anorexia nervosa. Mol Psychiatry 2014; 19: 1085–1094. 24514567 10.1038/mp.2013.187 PMC4325090

[bib36] Egan MF, Kojima M, Callicott JH, Goldberg TE, Kolachana BS, Bertolino A et al. The BDNF val66met polymorphism affects activity-dependent secretion of BDNF and human memory and hippocampal function. Cell 2003; 112: 257–269. 12553913 10.1016/s0092-8674(03)00035-7

[bib37] Mercader JM, Ribasés M, Gratacòs M, González JR, Bayés M, de Cid R et al. Altered brain-derived neurotrophic factor blood levels and gene variability are associated with anorexia and bulimia. Genes Brain Behav 2007; 6: 706–716. 17376155 10.1111/j.1601-183X.2007.00301.x

[bib38] Monteleone P, Maj M. Dysfunctions of leptin, ghrelin, BDNF and endocannabinoids in eating disorders: beyond the homeostatic control of food intake. Psychoneuroendocrinology 2013; 38: 312–330. 23313276 10.1016/j.psyneuen.2012.10.021

[bib39] Berridge KC, Robinson TE, Aldridge JW. Dissecting components of reward: "liking", "wanting", and learning. Curr Opin Pharmacol 2009; 9: 65–73. 19162544 10.1016/j.coph.2008.12.014 PMC2756052

[bib40] Berridge KC. The debate over dopamine's role in reward: the case for incentive salience. Psychopharmacology 2007; 191: 391–431. 17072591 10.1007/s00213-006-0578-x

