# Supplementary material for: Higher reward value of starvation imagery in anorexia nervosa and association with the Val66Met BDNF polymorphism
Source: Transl Psychiatry. 2016 Jun 7;6(6):e829–. doi: 10.1038/tp.2016.98 (PMC4931615; doi:10.1038/tp.2016.98)
Supplement: Supplementary Table 1 [file tp201698x1.pdf]

Supplementary Table 1. Electrophysiological response to weight stimuli (under normal and overweight) between patient with Anorexia Nervosa with and without medication

Visual stimuli	Responses		Sum of Squares	df	Mean Square	F	p
Underweight	SC +	Between groups	0.099	1	0.099	2.241	0.139
		Within	3.035	69	0.044		
		Total	3.133	70			
	SC amplitude	Between groups	0.144	1	0.144	0.905	0.345
		Within Groups	11.002	69	0.159		
		Total	11.147	70			
Normal weight	SC +	Between groups	0.015	1	0.015	0.091	0.763
		Within Groups	11.267	69	0.163		
		Total	11.282	70			
	SC amplitude	Between groups	0.093	1	0.093	0.087	0.769
		Within Groups	73.92	69	1.071		
		Total	74.013	70			
Overweight	SC +	Between groups	0.015	1	0.015	0.472	0.494
		Within Groups	2.125	69	0.031		
		Total	2.14	70			
	SC amplitude	Between groups	0.183	1	0.183	0.915	0.342
		Within Groups	13.595	68	0.2		
		Total	13.778	69			

SC+: Skin Conductance response (average frequency)

SC amplitude: Skin Conductance amplitude
