# Supplementary material for: Higher reward value of starvation imagery in anorexia nervosa and association with the Val66Met BDNF polymorphism
Source: Transl Psychiatry. 2016 Jun 7;6(6):e829–. doi: 10.1038/tp.2016.98 (PMC4931615; doi:10.1038/tp.2016.98)
Supplement: Supplementary Table 2 [file tp201698x2.pdf]

Supplementary Table 2. Emotional and electrophysiological response to underweight stimuli between patient with Anorexia Nervosa restricting type and patients having the binge eating/purging type

			Sum of Squares	df	Mean Square	F	p
Underweight	Feel task	Between groups	0.649	1	0.649	3.153	0.08
		Within Groups	14.203	69	0.206		
		Total	14.852	70			
	SC +	Between groups	.007	1	.007	.165	.686
		Within Groups	3.126	69	.045		
		Total	3.133	70			
	SC amplitude	Between groups	0.04	1	0.04	0.25	0.619
		Within Groups	11.106	69	0.161		
		Total	11.147	70			

SC+: Skin Conductance response (average frequency)
SC amplitude: Skin Conductance amplitude
