# Supplementary material for: Higher reward value of starvation imagery in anorexia nervosa and association with the Val66Met BDNF polymorphism
Source: Transl Psychiatry. 2016 Jun 7;6(6):e829–. doi: 10.1038/tp.2016.98 (PMC4931615; doi:10.1038/tp.2016.98)
Supplement: Supplementary Table 3 [file tp201698x3.pdf]

Supplementary table 3. ANCOVA of characteristics of the emotional and electrophysiological response to weight stimuli in Anorexia Nervosa and Healthy Control groups

Source	Stimuli	Dependent Variable	Type III Sum of Squares	df	Mean Square	F	p
Corrected Model	Underweight	Feel task	12.275	3	4.092	23.36	<0.001
		SC +	1.096	3	0.365	11.005	<0.001
		SC amplitude	2.626	3	0.875	4.283	<0.001
	Normal Weight	Feel task	9.473	3	3.158	11.264	<0.001
		SC +	0.415	3	0.138	1.074	0.364
		SC amplitude	2.018	3	0.673	0.788	0.162
	Overweight	Feel task	1.401	3	0.467	8.305	<0.001
		SC +	0.142	3	0.047	1.755	0.162
		SC amplitude	1.643	3	0.548	2.34	0.079
Intercept	Underweight	Feel task	316.027	1	316.027	1804.303	<0.001
		SC +	4.67	1	4.67	140.626	<0.001
		SC amplitude	28.095	1	28.095	137.481	<0.001
	Normal Weight	Feel task	277.013	1	277.013	988.111	<0.001
		SC +	4.913	1	4.913	38.176	<0.001
		SC amplitude	25.059	1	25.059	29.355	<0.001
	Overweight	Feel task	85.437	1	85.437	1518.923	<0.001
		SC +	3.923	1	3.923	145.016	<0.001
		SC amplitude	22.448	1	22.448	95.898	<0.001
Group	Underweight	Feel task	10.239	1	10.239	58.457	<0.001
		SC +	8.102	1	8.102	28.901	<0.001
		SC amplitude	0.845	1	0.845	25.455	0.001
	Normal Weight	Feel task	8.102	1	8.102	28.901	<0.001
		SC +	0.115	1	0.115	0.897	0.346
		SC amplitude	0.043	1	0.043	0.05	0.824
	Overweight	Feel task	1.333	1	1.333	23.702	<0.001
		SC +	0.073	1	0.073	2.682	0.105
		SC amplitude	0.975	1	0.975	4.167	0.044
Genotype	Underweight	Feel task	0.237	1	0.237	1.351	0.248
		SC +	0.064	1	0.064	1.925	0.169
		SC amplitude	0.362	1	0.362	1.771	0.187
	Normal Weight	Feel task	0.332	1	0.332	1.185	0.279
		SC +	0.144	1	0.144	1.116	0.294
		SC amplitude	0.684	1	0.684	0.801	0.373
	Overweight	Feel task	0.236	1	0.236	4.19	0.044
		SC +	0.039	1	0.039	1.442	0.233
		SC amplitude	0.072	1	0.072	0.31	0.579
Group * Genotype	Underweight	Feel task	0.173	1	0.173	0.989	0.323
		SC +	0.038	1	0.038	1.149	0.287
		SC amplitude	0.177	1	0.177	0.868	0.354
	Normal Weight	Feel task	0.019	1	0.019	0.068	0.794
		SC +	0.008	1	0.008	0.064	0.801
		SC amplitude	0.194	1	0.194	0.227	0.635
	Overweight	Feel task	0.184	1	0.184	3.27	0.074
		SC +	0,00	1	0,00	0.006	0.938
		SC amplitude	0.266	1	0.266	1.138	0.289

ANCOVA, made with general linear model in SPSS
SC+: Skin Conductance response (average frequency)
SC amplitude: Skin Conductance amplitude
