# Supplementary material for: Higher reward value of starvation imagery in anorexia nervosa and association with the Val66Met BDNF polymorphism
Source: Transl Psychiatry. 2016 Jun 7;6(6):e829–. doi: 10.1038/tp.2016.98 (PMC4931615; doi:10.1038/tp.2016.98)
Supplement: Supplementary Table 4 [file tp201698x4.pdf]

Supplementary Table 4. Correlations between emotional and electrophysiological response to underv
clinical dimensions in Anorexia Nervosa patients

Variables		Feel task	SC+
Current body mass index (kg/m²)	pearson correlation	0.14	-.144
	p value	0.409	0.229
Lowest lifetime body mass index (kg/m²)	pearson correlation	0.06	-.088
	p value	0.61	0.467
Number of hospitalizations	pearson correlation	0.15	0.06
	p value	0.23	0.622
Age at onset (years)	pearson correlation	0.009	-.050
	p value	0.94	0.681
Duration of illness (years)	pearson correlation	-.074	0.03
	p value	0.54	0.805
Body Shape Questionnaire scores	pearson correlation	0.47	0.08
	p value	<0.001	0.538

SC+: Skin Conductance response (average frequency)
SC amplitude: Skin Conductance amplitude

weight stimuli and

SC amplitude
-.213
0.074
-.178
0.138
0.23
0.059
-.209
0.08
-.179
0.136
0.11
0.399
