# Supplementary material for: Higher reward value of starvation imagery in anorexia nervosa and association with the Val66Met BDNF polymorphism
Source: Transl Psychiatry. 2016 Jun 7;6(6):e829–. doi: 10.1038/tp.2016.98 (PMC4931615; doi:10.1038/tp.2016.98)
Supplement: Supplementary Table 5 [file tp201698x5.pdf]

Supplementary Table 5. Linear regression model for the Anorexia Nervosa patients group, studying the relationship between SCR response during processing of the underweight stimuli, the Met allele of the BDNF, and clinical criteria of severity (age, current BMI, minimal BMI, psychotropic medication, subtype of anorexia nervosa, number of hospitalizations, duration of illness and severity score of the Body Shape Questionnaire)

Model	Regression coefficient	Standard error	t	p
(Constante)	0.792	0.305	2.6	0.012
Genotype	-0.157	0.057	-2.729	0.008
Age	-0.001	0.003	-0.319	0.751
Current BMI (kg/m^2)	-0.006	0.014	-0.438	0.663
Lowest lifetime BMI (kg/m^2)	-0.002	0.017	-0.122	0.904
Medication	0.057	0.06	0.952	0.345
Subtype of Anorexia Nervosa	-0.039	0.057	-0.697	0.489
Number of hospitalizations	0.002	0.013	0.159	0.875
Duration of illness (years)	0	0	0.887	0.379
Body Shape Questionnaire scores	0	0.001	0.201	0.841
