# Supplementary material for: Higher reward value of starvation imagery in anorexia nervosa and association with the Val66Met BDNF polymorphism
Source: Transl Psychiatry. 2016 Jun 7;6(6):e829–. doi: 10.1038/tp.2016.98 (PMC4931615; doi:10.1038/tp.2016.98)

Supplementary Figure 2. Average amplitude of SCR to visual stimuli (underweight, normal weight and overweight) in patients with Anorexia Nervosa (N=71) and in Healthy Controls (N=20).
*p<0,05. *inter group significant differences

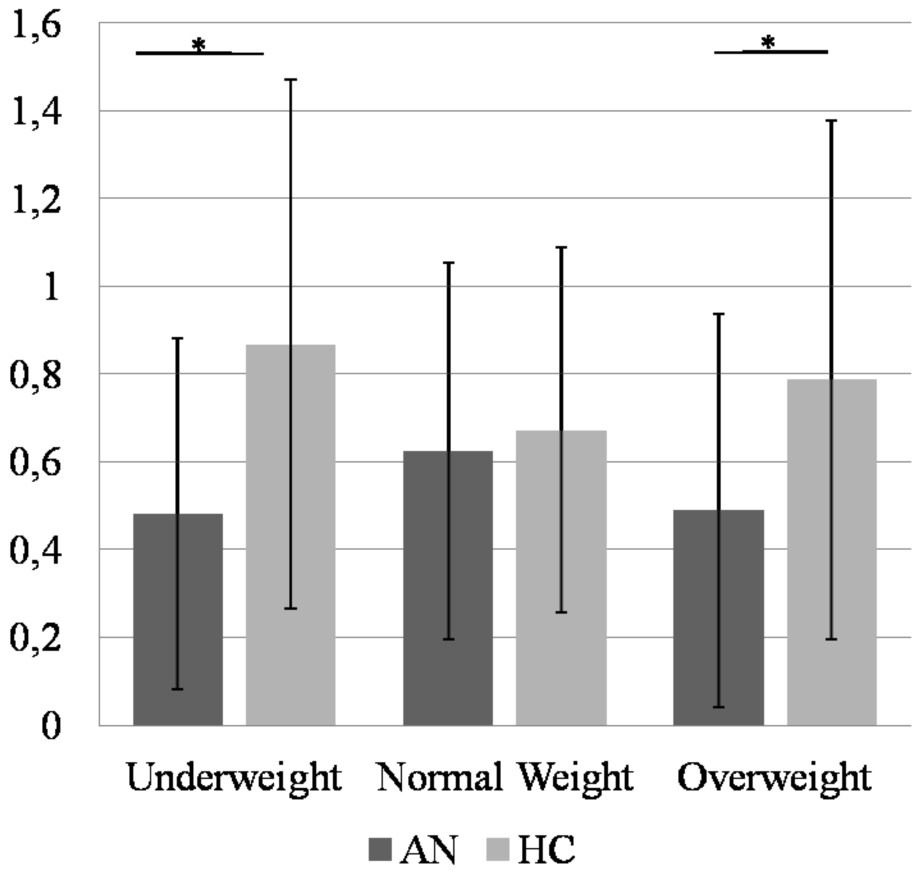

Supplement: Supplementary Figure 2 [file tp201698x7.pdf]
